# Supplementary material for: A graph-based cell tracking algorithm with few manually tunable parameters and automated segmentation error correction
Source: PLoS One. 2021 Sep 7;16(9):e0249257. doi: 10.1371/journal.pone.0249257 (PMC8423278; doi:10.1371/journal.pone.0249257)
Supplement: S1 Fig — (PDF) [file pone.0249257.s001.pdf]

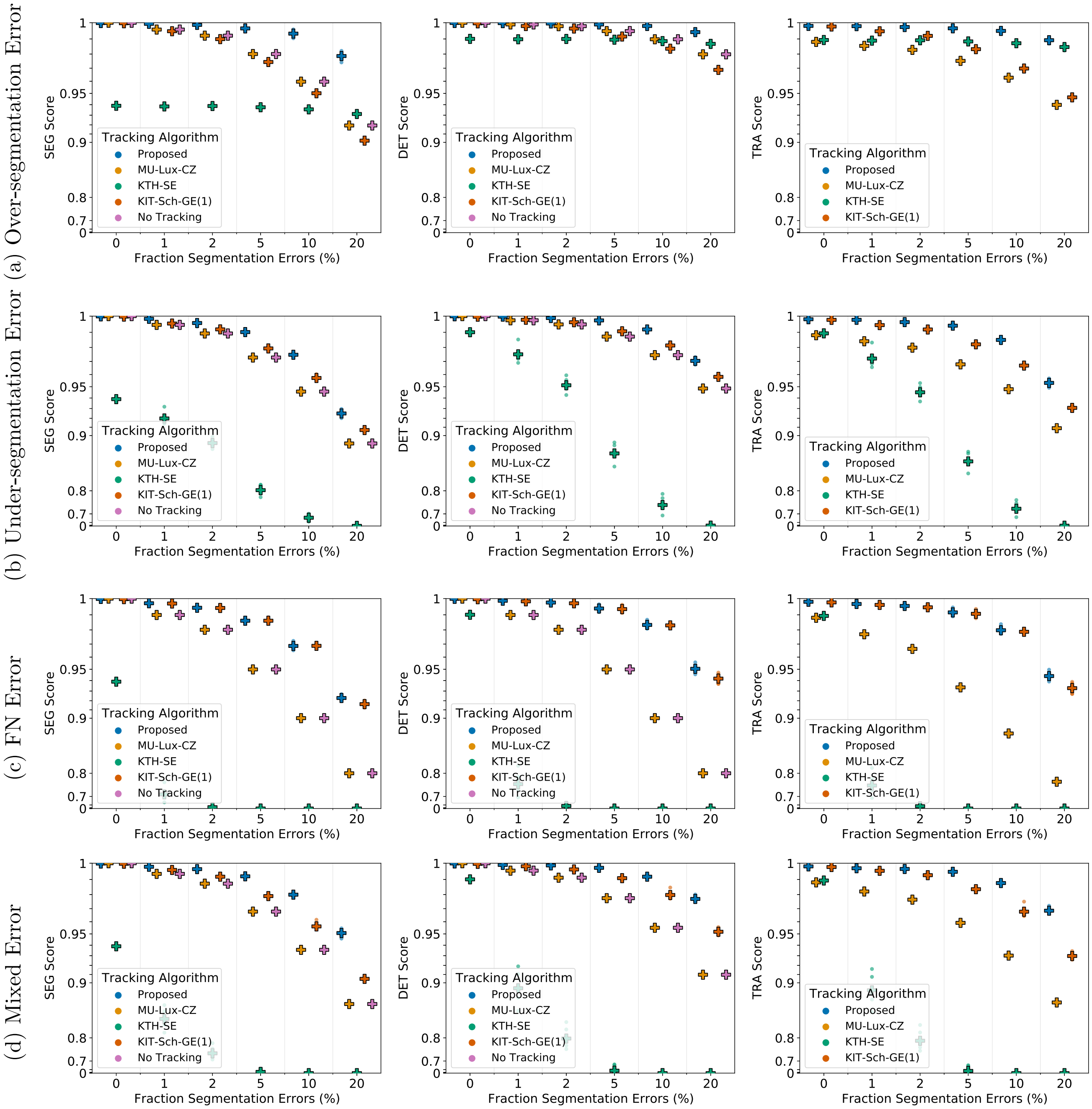

Fig. S1. Influence of the post-processing on Fluo-N2DH-SIM+ 02. Scores of a single run are shown as circles, while + shows a CTC measure score averaged over $N = 5$ runs. Per run a fixed fraction of ground truth segmentation masks is modified randomly to simulate segmentation errors. "untangle" refers to the untangling step, which transforms the tracking graph such that each track has at most one predecessor and two successors, whereas "masks" refers to adding missing segmentation masks. Over lined post-processing steps $\overline{(\ldots)}$ indicate that the post-processing step is missing.
