# Supplementary material for: A graph-based cell tracking algorithm with few manually tunable parameters and automated segmentation error correction
Source: PLoS One. 2021 Sep 7;16(9):e0249257. doi: 10.1371/journal.pone.0249257 (PMC8423278; doi:10.1371/journal.pone.0249257)
Supplement: S4 Fig — (PDF) [file pone.0249257.s004.pdf]

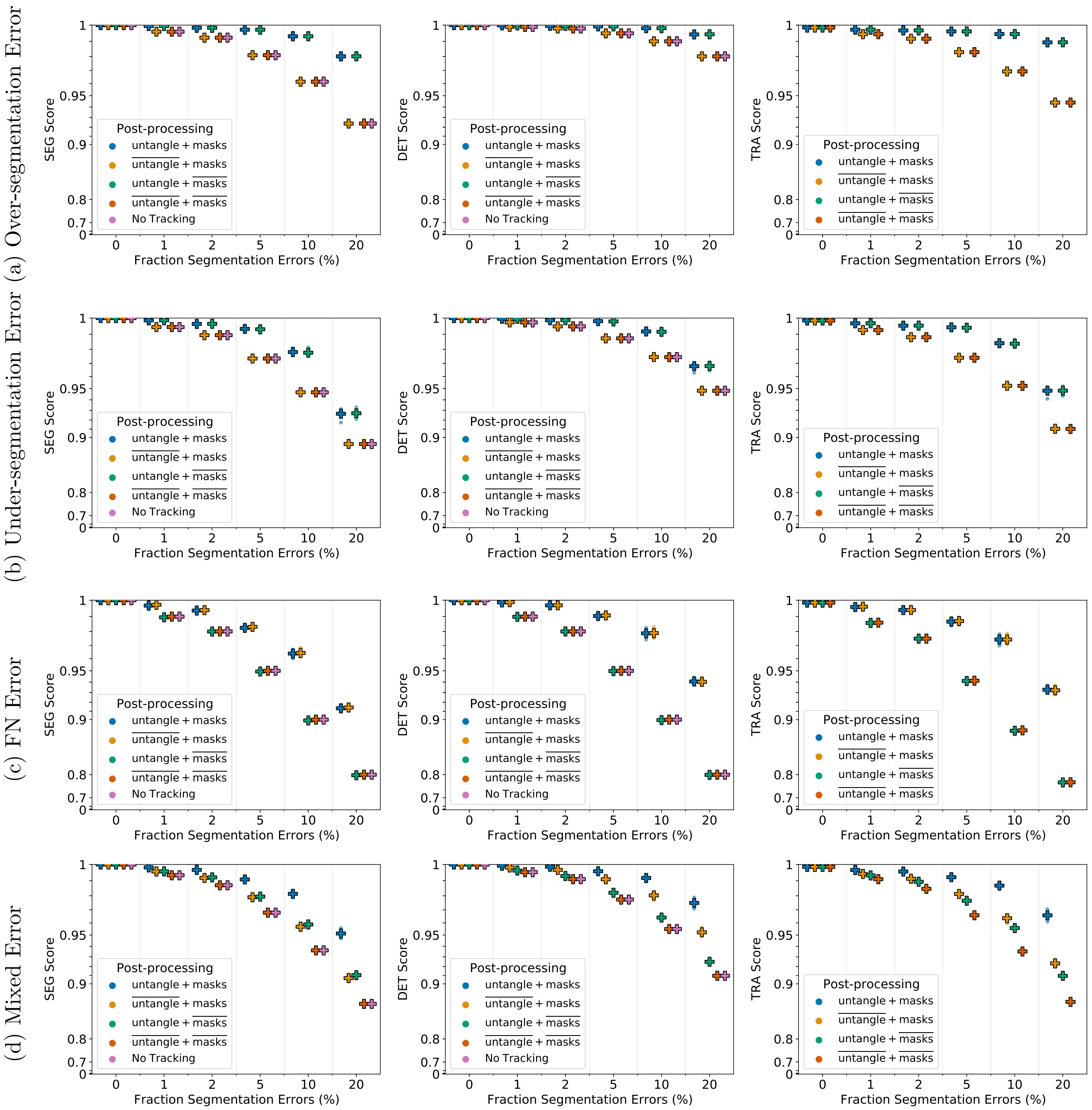

Fig. S4. Comparing tracking algorithms on Fluo-N3DH-SIM+ 02. Shown are the CTC measure scores DET, SEG, and TRA of tracking algorithms on 2D data set Fluo-N3DH-SIM+ 02 when provided with the same erroneous segmentation data. Scores of a single run are shown as circles, while + shows a CTC measure score averaged over $N = 5$ runs.
