# Supplementary material for: A graph-based cell tracking algorithm with few manually tunable parameters and automated segmentation error correction
Source: PLoS One. 2021 Sep 7;16(9):e0249257. doi: 10.1371/journal.pone.0249257 (PMC8423278; doi:10.1371/journal.pone.0249257)
Supplement: S1 Table — (PDF) [file pone.0249257.s005.pdf]

Table S1. Run-times of tracking algorithms on image sequences 02. Run times of the tracking algorithms on 2D and 3D data sets, image sequence 02, when provided with perfect ground truth (GT) segmentation as well as when provided with erroneous segmentation data.

**Tracking Algorithm**	**Data Sets**			
	Fluo-N2DH-SIM+ 02		Fluo-N3DH-SIM+ 02	
	GT	Erroneous	GT	Erroneous
Proposed	28.85 s	29.19 s	1206.05 s	1312.72 s
MU-Lux-CZ	45.15 s	46.12 s	2312.94 s	2323.22 s
KTH-SE	49.71 s	48.85 s	477.17 s	375.26 s
KIT-Sch-GE	20.38 s	31.27 s	1160.88 s	1678.79 s
