# Supplementary material for: A graph-based cell tracking algorithm with few manually tunable parameters and automated segmentation error correction
Source: PLoS One. 2021 Sep 7;16(9):e0249257. doi: 10.1371/journal.pone.0249257 (PMC8423278; doi:10.1371/journal.pone.0249257)
Supplement: S1 File — (PDF) [file pone.0249257.s006.pdf]

Data Availability

Tracking Results on Data with Simulated Segmentation Errors

The synthetically simulated segmentation errors can be reproduced by running the script `create_synth_segm_data.py` from our repository `https://git.scc.kit.edu/KIT-Sch-GE/2021-cell-tracking`.
All tracking results – sequences of tracked masks, the lineage and the CTC metrics DET,SEG, and TRA – are provided in the repositories `https://zenodo.org/record/5227595` and `https://zenodo.org/record/5227610`.

Tracking Results on the CTC

All tracking results from our proposed tracking algorithm on the CTC data can be reproduced by running our tracking code by calling `run_tracking.py` with the set default parameters from our repository. The default parameters are the same parameters as used for the evaluation on simulated erroneous segmentation data and are stated in the parameter selection section of the paper.
