# Supplementary material for: A graph-based cell tracking algorithm with few manually tunable parameters and automated segmentation error correction
Source: PLoS One. 2021 Sep 7;16(9):e0249257. doi: 10.1371/journal.pone.0249257 (PMC8423278; doi:10.1371/journal.pone.0249257)
Supplement: Attachment — Submitted filename: Response to Reviewers.docx [file pone.0249257.s007.docx]

Dear Konradin Metze,

thank you very much for editing our revised manuscript entitled “A graph-based cell tracking algorithm with few manually tunable parameters and automated segmentation error correction”. Based on the reports of the reviewers, we extended the analysis of our proposed tracking algorithm by adding additional measures and evaluated the algorithm on diverse 2D and 3D data sets. Moreover, we redesigned our figures to ease the readers understanding of the tracking pipeline. As one reviewer pointed out the complex notation in the paper we carefully revised our notation to reduce confusion. Meanwhile, we submitted and received the results of our contribution to the 6^th^ ISBI Cell Tracking Challenge and added them, following the suggestion of the reviewers to evaluate on additional data sets. The changes to the initially submitted manuscript are highlighted in blue (added) and red (removed) in the marked-up version. We expanded the supplementary material with additional results as well. Please find below our prepared answers for the reviewers.

Yours sincerely,

Katharina Löffler

--------------------------

Reviewer: 1

Dear Reviewer,

Thank you for sending us your comments and suggestions. We are pleased to read, that you find our algorithm interesting. We have also prepared answers to your comments:

“1. The algorithm sections are quite difficult to read. The notations are very complex. It feels like each set of equations were using very non-overlapping sets of symbols that represent different things. It is true that different sets of equations described different steps/components of the algorithm. If the notations can be significantly simplified, it will be a lot easier for readers to follow this paper and appreciate the algorithmic designs.”

Answer:

We carefully revised parts of our notation to avoid confusion due to the naming of indices and made the naming of variables which are optimized more consistent. The symbol t now always indicates the time. Previously t was used for source (t+) & sink (t⁻) nodes and for time points. This allowed us to replace the variable k used in the node indices (now always u_*,t_ instead of u_*,k_) and to avoid confusions with node naming, now referred to as source (q+) and sink (q-). The notation for optimization variables is now more consistent. The flow variables are renamed to z^f^ (f before) and coupled flow variables to z^f^_jl_ (f_jl_.before) The optimization variables in the untangling problem are now: z^e^_pn_ (e_ij_ before), z_r_^m^ (m_l_ before), and z^s^_n_ (s_i_ before). We also adapted the notation of the cost functions for the untangling problem: c^e^_pn_ (c(e_ij_) before), c^m^_r_ (c(m_l_) before), and c^s^_n_ (c(s^i^) before). Moreover, we renamed some indices to avoid confusion between the indices of the two different optimization problems.

„2. The figures are quite repetitive in some sense, the same graph topology appearing in 4 different figures. Again, I understand that they convey different information on different steps of the algorithm, but I would be better to consolidate them into one figure with multiple sub-figures, so that it is easier for readers to understand the whole flow. “

Answer:

We agree that the overall number of figures can be reduced to improve the flow for the readers. We adapted the figures accordingly – We kept Figure 1 for conceptual overview. Figures 2&3 are combined so the graph example and the costs are shown in a single figure. We combined Figures 4&5 now showing the extracted features and how they can be used to match correctly and erroneously segmented objects. Figures 5&6 are combined as well, showing the untangling operations and the untangling problem.

Yours sincerely,

Katharina Löffler

--------------------------

Reviewer: 2

Dear Reviewer,

We are excited to read that you think our paper, which proposes a cell tracking algorithm, has merit to be published in PLOS ONE. We have also prepared answers to your comments:

“1. The algorithms seems to work on separated cell (cell lines only), and not on tissues (incompletely acquired objects). This should be discussed.”

Answer:

Usually the problem on tissues is more a segmentation issue, as these cells are rather constrained in their position. Hence, most tissue analysis problems (especially in pathology) do not require a tracking analysis. In contrast, on cell data sets where cells can move freely the tracking is more challenging as now the tracking algorithm needs to handle large shifts in the cell position as well as segmentation errors, e.g. due to low resolution in the z-axis. However, our tracking algorithm can handle a variety of cell types (shapes, sizes and textures) and imaging acquisition techniques as shown in the added Table 3. The main prerequisite is a segmentation of the objects to track.

“2. What kind of principle segmentation algorithm has been used (dynamic, static, Otsu, …) etc. or texture / transformation based methods (Fourier, Laplace, …)?”

Answer:

In our initial manuscript we investigated how tracking algorithms handle erroneous segmentation data. Therefore, we simulated segmentation errors based on a provided, perfect ground truth. As the data sets Fluo-N2DH-SIM+ and Fluo-N3DH-SIM+ from the Cell Tracking Challenge are synthetic data sets, their ground truth segmentation masks are generated synthetically as well. Thus, no segmentation algorithm was needed. Now, we have added an evaluation of our tracking algorithm on a total of 15 data sets of the Cell Tracking Challenge, where we used a deep learning based segmentation approach. The new Table 3 shows that such a combination is very competitive. However, any segmentation approach could be combined with our proposed tracking step.

“3. Are different cell types included in this study?”

Answer:

We extended our analysis and applied the tracking algorithm on a total of 15 different cell data sets, showing different cell types imaged in 2D or 3D as well as different imaging conditions – Fluorescently counterstained, Bright Field, Phase Contrast, and Differential Interference Contrast. The results are shown in Table 3. The discussion has been adapted accordingly.

“4. The authors should clearly distinguish the different mandatory compartments of the algorithms, namely (image acquisition (including image quality), object detection (differentiation potential Object / background), and object identification (object features) and classification (object class) for additional analysis (for example texture).
For example see and cite) (KAYSER, Klaus; BORKENFELD, Stephan; KAYSER, Gian. Digital Image Content and Context Information in Tissue-based Diagnosis. Diagnostic Pathology, [S.l.], v. 4, n. 1, dec. 2018. ISSN 2364-4893; GÖRTLER, Jürgen et al. Cognitive Algorithms and digitized Tissue – based Diagnosis. Diagnostic Pathology, [S.l.], v. 3, n. 1, july 2017. ISSN 2364-4893.“

Answer:

As we propose the tracking step for a tracking by detection pipeline, a segmentation method – for example deep learning based – needs to be selected by the user. We agree that our tracking algorithm has to be embedded into more complex analysis pipelines, but we think that a detailed discussion of complete pipelines is beyond the scope of the paper.

“5. A few words should be addressed to future development (understanding) how structures 8cells) and functions (movements) might depend on each other. For example see and cite (Kayser, Klaus, Borkenfeld, Stephan, Fang, Wei-Kleiner, Kayser, Gian: Digital Pathology Where did – do – will you go? Content and Context Analysis of Communication in Digital Pathology. <https://doi.org/10.17629/www.medical-journal-of-virtual-science.de-2021-1:2>.“

Answer:

Thank you for suggesting this interesting paper. In our opinion, it is definitely a future task to correlate structure and behavior. We assume, however, that pathology is here a very ambitious field because most samples cannot be observed over time by an in vivo imaging approach. While we see a clear motivation for segmentation approaches on pathological images, we find it challenging to see the connection between the suggested paper and our proposed cell tracking method, as usually pathological images capture immobile cells. Thus, we have decided not to cite it.

Yours sincerely,

Katharina Löffler

--------------------------

Reviewer: 3

Dear Reviewer,

Thank you for your comments and suggestions. We are pleased to read that you appreciated the overall presentation of the content. We have also prepared answers to your suggestions:

“The drawback of the presentation is that the algorithm was only tested on two of 19 available datasets from the CTC. Another disadvantage is that only the tracking accuracies TRA are presented. With this minimal comparison, the evaluation of the proposed new algorithm cannot be performed satisfactorily.
Therefore, I would suggest adding further samples and adding the values for segmentation accuracies, detection accuracies, cell segmentation benchmarks and cell tracking benchmarks.“

Answer:

We extended our initial analysis on synthetically degraded segmentation data by computing the DET and SEG scores from the CTC as well. We have updated the corresponding figures. In addition, we added our results from the Cell Tracking Benchmark on this year’s CTC, where we participated on a diverse set of 2D and 3D data sets (15 in total). To evaluate the performance of our algorithm, we used the same manually tunable parameter setting on all submitted 2D and 3D data sets. We think the added results emphasize the capabilities of our tracking algorithm to perform competitively on a diverse set of 2D and 3D data sets without the need of fine tuning.

Yours sincerely,

Katharina Löffler
