# Supplementary material for: A graph-based cell tracking algorithm with few manually tunable parameters and automated segmentation error correction
Source: PLoS One. 2021 Sep 7;16(9):e0249257. doi: 10.1371/journal.pone.0249257 (PMC8423278; doi:10.1371/journal.pone.0249257)
Supplement: Attachment — Submitted filename: Response to Reviewers.docx [file pone.0249257.s008.docx]

Dear Konradin Metze,

thank you very much for editing our revised manuscript entitled “A graph-based cell tracking algorithm with few manually tunable parameters and automated segmentation error correction”. We assume that our background and the one of reviewer 2 are quite different, resulting in an unfortunate misunderstanding on our side on how to interpret the requested adaptions. We hope to clarify all misunderstanding by adding further explanations and references to the paper. The changes to the compared to the last revision are highlighted in blue (added) and red (removed) in the tracked changes manuscript version.

Yours sincerely,

Katharina Löffler

--------------------------

Reviewer: 1

Dear Reviewer,

Thank you for your helpful suggestions to improve the notation and graphics.

Yours sincerely,

Katharina Löffler

--------------------------

Reviewer: 2

Dear Reviewer,

We are sorry to read that you feel we are unwilling to accept your suggested modifications. We might have misunderstood parts of your suggestions and thought that you just wanted some further explanations, which we gave to you. However, we see that the paper also needed some further editing. We hope that the added comments and modifications on the paper can help to resolve this misunderstanding. Please find below answers to your comments.

-----

We think the first point 1 from your first revision was due to a misunderstanding of the scope and the content of the paper, which we decided to clarify in the rebuttal letter only:

“1. The algorithms seems to work on separated cell (cell lines only), and not on tissues (incompletely acquired objects). This should be discussed.”

Answer:

As we proposed a tracking algorithm and not a segmentation algorithm our focus lies on data sets showing moving cells. Therefore, we used data sets from the in the cell tracking community well-established Cell Tracking Challenge to evaluate and compare our cell tracking algorithm.

Unfortunately, we have trouble understanding on what is meant by incompletely acquired objects in the scope of a tracking problem.

-----

All other suggestions from the first revision have now been added to the paper (or were already resolved but not clearly indicated in our rebuttal letter). We would like to summarize how we addressed your required modifications / recommendation by providing quotes from the paper:

“2. What kind of principle segmentation algorithm has been used (dynamic, static, Otsu, …) etc. or texture / transformation based methods (Fourier, Laplace, …)?”

Answer:

We now added the text passage (page 3, lines 81-84 in the tracked changes manuscript version)

“In this section, we describe our tracking algorithm, which is able to process 2D and 3D image sequences, in more detail. To create a tracking by detection algorithm, our proposed tracking algorithm can be combined with an arbitrary segmentation algorithm, which predicts instance segmentation masks.”

to clarify that our tracking algorithm can be combined with any segmentation approach.

Moreover, for our participation on the 6^th^ edition of the CTC (results in Table 3) we selected a deep learning based segmentation (page 16, lines 541-543 in the tracked changes manuscript version)

“For segmentation, we chose a deep learning based segmentation approach which utilizes cell and neighbor distances [9, 57]. The derived segmentation masks and the raw image sequence were fed into our tracking algorithm. ”

Since we added this text passage already in the last revision and only changes to that revision are highlighted in blue, that change is not highlighted.

For our analysis on how different tracking algorithms behave, when provided with erroneous segmentation data, we used the perfect, segmentation masks from the data sets Fluo-N2DH-SIM+ and Fluo-N3DH-SIM+ which are provided with the data. These two data sets are synthetically generated; thus no additional segmentation is needed to generate the used ground truth segmentation masks. We emphasize that these two data sets are synthetic by adding (page 13, lines 429-430 in the tracked changes manuscript version)

“Both cell data sets show *synthetically generated* human leukemia cells, where Fluo-N2DH-SIM+ is a 2D data set and Fluo-N3DH-SIM+ is a 3D data set.”

-----

“3. Are different cell types included in this study?”

Answer:

We evaluated on 15 different cell data sets, please see Table 3 (page 17) for the different examined cell types. We added the table already in the last revision to answer your question.

-----

“4. The authors should clearly distinguish the different mandatory compartments of the algorithms, namely (image acquisition (including image quality), object detection (differentiation potential Object / background), and object identification (object features) and classification (object class) for additional analysis (for example texture).
For example see and cite) (KAYSER, Klaus; BORKENFELD, Stephan; KAYSER, Gian. Digital Image Content and Context Information in Tissue-based Diagnosis. Diagnostic Pathology, [S.l.], v. 4, n. 1, dec. 2018. ISSN 2364-4893; GÖRTLER, Jürgen et al. Cognitive Algorithms and digitized Tissue – based Diagnosis. Diagnostic Pathology, [S.l.], v. 3, n. 1, july 2017. ISSN 2364-4893.“

Answer:

We added a paragraph to clarify the additional components needed to create a tracking by detection approach. Furthermore, we emphasized that the tracking actually is a step in an image analysis pipeline (page 3, lines 81-86 in the tracked changes manuscript version):

“In this section, we describe our tracking algorithm, which is able to process 2D and 3D image sequences, in more detail. To create a tracking by detection algorithm, our proposed tracking algorithm can be combined with an arbitrary segmentation algorithm, which predicts instance segmentation masks. Moreover, the tracking can be included in a full image analysis pipeline which typically consists of sample preparation and imaging, cell segmentation, cell tracking, and subsequent analysis [51, 52].”

Therefore, we added also the new references:

[51] Megason SG, Fraser SE. Imaging in systems biology. Cell. 2007;130(5):784–795.doi:10.1016/j.cell.2007.08.031.52.

[52] Eliceiri KW et al. Biological imaging software tools. Nature Methods. 2012;9(7):697–710.doi:10.1038/nmeth.2084

-----

“5. A few words should be addressed to future development (understanding) how structures 8cells) and functions (movements) might depend on each other. For example see and cite (Kayser, Klaus, Borkenfeld, Stephan, Fang, Wei-Kleiner, Kayser, Gian: Digital Pathology Where did – do – will you go? Content and Context Analysis of Communication in Digital Pathology. <https://doi.org/10.17629/www.medical-journal-of-virtual-science.de-2021-1:2>.“

Answer:

To put our work into the greater picture, we added a paragraph to the conclusion (page 19, lines 618-622 in the tracked changes manuscript version):

“We envision that the steady improvement of automated cell tracking approaches concerning the accuracy, run time, and ease of applicability will lead to powerful tools to analyze cell behavior quantitatively. The derived insights on the cell behavior can then help to deepen our understanding of the mechanisms influencing cell migration or, for instance, how cell migration and the formation of structures depend on each other.”

-----

Concerning the last point of your second review	:

“There are some major inconsistencies, for example 'ground truth does not need segmentation' which need essential explanation.
Therefore, I cannot recomment the article for publication”

Answer:

We are highly motivated to further improve the quality of our paper and therefore are looking forward to your feedback on major inconsistencies. Unfortunately, as only one of them is provided here we are only able to resolve this one.

We would like to refer to (page13, lines 426-428 of the tracked changes manuscript version):

“We select the cell data sets Fluo-N2DH-SIM+ and Fluo-N3DH-SIM+ from the CTC [5, 50] for evaluation, as they are publicly available and provide a fully annotated ground truth, i.e. segmentation masks are given for all cells as well as the cell lineage.”

In which we explain what kind of ground truth data is available; all segmentation masks as well as the lineage are provided by the two selected CTC data set. As the data sets are simulated, no additional segmentation algorithm is needed. We tried to emphasize the synthetic nature of the data set by making the following adaption (page 13, lines 429-430 in the tracked changes manuscript version):

“Both cell data sets show *synthetically generated* human leukemia cells, where Fluo-N2DH-SIM+ is a 2D data set and Fluo-N3DH-SIM+ is a 3D data set.”

To evaluate how different tracking algorithms handle different types of segmentation errors, we then modify these provided segmentation masks to model segmentation errors (page 14, lines 433-456 of the tracked changes manuscript version).

We hope, that in the new manuscript there is no more room for confusion. Thank you for your helpful suggestions to improve this manuscript.

Yours sincerely,

Katharina Löffler

------------------

Reviewer 3:

Dear Reviewer,

Thank you for your suggestions to include additional metrics and strengthening the results section by evaluating on more data sets.

Yours sincerely,

Katharina Löffler
