# Supplementary material for: A graph-based cell tracking algorithm with few manually tunable parameters and automated segmentation error correction
Source: PLoS One. 2021 Sep 7;16(9):e0249257. doi: 10.1371/journal.pone.0249257 (PMC8423278; doi:10.1371/journal.pone.0249257)
Supplement: Attachment — Submitted filename: Response to Reviewers.docx [file pone.0249257.s009.docx]

Dear Konradin Metze,

thank you very much for your patience and time in editing our revised manuscript entitled “A graph-based cell tracking algorithm with few manually tunable parameters and automated segmentation error correction”. We hope to clarify the remaining concerns by discussing the influence of image quality and temporal resolution on the segmentation and tracking performance in the discussion section and added further references. The changes compared to the last revision are highlighted in blue (added) in the tracked changes manuscript version.

Yours sincerely,

Katharina Löffler

--------------------------

Reviewer: 2

Dear Reviewer,

Thank you very much for your patience and time in reviewing our manuscript. Please find below answers to your comments.

“There remains still a principle problem, which the authors should clarify: They state, that their tracking algorithm is not dependent upon the segmentation algorithm. That is only true, if the background remains the same during the tracking process, an assumption that is quite unlikely in reality. Examples are, if additional artifacts appear or if the tracked individuals confuse, or if the distance between different objects shrinks to zero? This is a major problem in graph theory too; its application requires a 'none-zero distance' between different nodes (or non-zero edges). Therefore, I propose again to discuss briefly the aspect of image quality and its impact on any derived action and to cite the given references. Herein I repeat my opinion. I cannot propose an article for publication which disregards important practical and theoretical aspects.”

Answer:

To discuss the dependence of the segmentation and tracking performance on the image quality, we added the following paragraph [pages 18-19 lines 596-606]:

“We would like to emphasize that the tracking performance depends on the instance segmentation which in turn depends on the image quality. To reduce the dependence on the image quality, the image quality can be improved substantially by applying image restoration methods before segmentation [58,59]. In addition, instance segmentation approaches applicable to a broad range of imaging conditions exist [60]. While our tracking approach can be combined with an arbitrary instance segmentation approach, different instance segmentation approaches can be prone to different types and quantities of segmentation errors. Our results on simulated, erroneous segmentation data show, that our tracking algorithm can correct certain types of randomly occurring segmentation errors, however, with decreasing segmentation quality the tracking quality decreases as well. “

Moreover, we now discussed the influence of the temporal resolution on the tracking performance as well as sketched a limitation of our tracking algorithm [page 19 lines 607-618]:

“In general, the tracking performance also depends on the temporal resolution of the image sequence. If the temporal resolution is high with respect to the cell movements -- cell movements are small between frames with respect to the cell size -- simple, nearest neighbor assignment is sufficient [61]. However, when the temporal resolution is restricted, e.g. to avoid photodamage, large cell movements between successive frames are possible. To assign the segmented cells correctly, more advanced approaches, such as graph-based approaches, are needed. As the results of the CTC show, our position-based costs perform well on a broad set of real world cell data sets, however, there are scenarios which will result in wrong assignments. For instance, consider two cells at time point t which have swapped their positions at time t+1, which is impossible to detect using position-based costs. To resolve such cases, the tracking costs can be adapted using more complex features based on texture or morphology of single cells [25,30,37].”

To support the dependence of segmentation and tracking performance on the image quality and the dependence of the tracking on the temporal resolution of the image sequence we added the following references:

[58] Weigert M, Schmidt U, Boothe T, Müller A, Dibrov A, Jain A, et al. Content-aware image restoration: Pushing the limits of fluorescence microscopy. Nature Methods. 2018;15(12):1090–1097. doi:10.1038/s41592-018-0216-7.

[59] Belthangady C, Royer LA. Applications, promises, and pitfalls of deep learning for fluorescence image reconstruction. Nature Methods. 2019;16(12):1215–1225.doi:10.1038/s41592-019-0458-z.

[60] Caicedo JC, Goodman A, Karhohs KW, Cimini BA, Ackerman J, Haghighi M, et al. Nucleus segmentation across imaging experiments: the 2018 Data Science Bowl. Nature Methods. 2019;16(12):1247–1253. doi:10.1038/s41592-019-0612-7.

[61] Yang FW, Tomášová L, Guttenberg Zv, Chen K, Madzvamuse A. Investigating optimal time step intervals of imaging for data quality through a novel fully-automated cell tracking approach. Journal of Imaging. 2020;6(7). doi:10.3390/jimaging6070066.

We hope the added paragraphs and references resolve the remaining remarks.

Yours sincerely,

Katharina Löffler

------------------
